# 2807. Using Less of Our Big Guns: Vancomycin De-Escalation Using Methicillin-Resistant *Staphylococcus aureus* Nasal Swab Screening Protocol

**DOI:** 10.1093/ofid/ofad500.2418

**Published:** 2023-11-27

**Authors:** Petchpailin D Sittirat, Sarah J Choi, Daniel Cain, Sidart Pradeep, Alisha M Jain, Nikitha Bommisetty, Sneha Kalluri, Bryan Youree, Vivek Ramarathnam, Jamil A Abbasi, Alexander Bastidas, Rick Myers, Sunaina Rao, Theresa Yarger, Sommer Smith, Adrienne Carr, Shovendra Gautam

**Affiliations:** Baylor Scott and White All Saints, Fort Worth, Texas; Baylor Scott and White All Saints, Fort Worth, Texas; Baylor Scott and White All Saints, Fort Worth, Texas; Baylor Scott and White All Saints, Fort Worth, Texas; Baylor Scott and White All Saints, Fort Worth, Texas; Baylor Scott and White All Saints, Fort Worth, Texas; Baylor Scott and White All Saints, Fort Worth, Texas; Baylor Scott and White All Saints, Fort Worth, Texas; Baylor Scott and White All Saints, Fort Worth, Texas; Baylor Scott and White All Saints, Fort Worth, Texas; Baylor Scott and White All Saints, Fort Worth, Texas; Baylor Scott and White All Saints, Fort Worth, Texas; Baylor Scott and White All Saints, Fort Worth, Texas; Baylor Scott and White All Saints, Fort Worth, Texas; Baylor Scott and White All Saints, Fort Worth, Texas; Baylor Scott and White Health, Keller, Texas; Baylor Scott and White All Saints Medical Center- Fort Worth, Fort Worth, Texas

## Abstract

**Background:**

Empiric parenteral vancomycin is crucial to early sepsis treatment due to its efficacy against organisms resistant to other antibiotics, including Methicillin-resistant *Staphylococcus aureus* (MRSA). Reliance on broad spectrum antibiotics has contributed to antibiotic resistant organisms and nearly 5 million deaths in 2019 [1]. The goal of this study was to improve antibiotic stewardship by using MRSA nasal swab screening to expedite parenteral vancomycin discontinuation. Total hospital and ICU length of stay, sepsis and severe sepsis incidence, *Clostridium difficile* incidence rates and institutional vancomycin usage were also measured.

**Methods:**

This project is a retrospective observational study on patients admitted to the ICU and placed on parenteral vancomycin over a 31-month period. Using the classic Plan-Do-Study-Act cycle, a standing antibiotic de-escalation protocol (the “Protocol”) was created which called for a pharmacist to order a nasal MRSA swab on the patient within 48 hours of vancomycin initiation. The critical care team used these results to decide whether to continue vancomycin.

**Figure:**
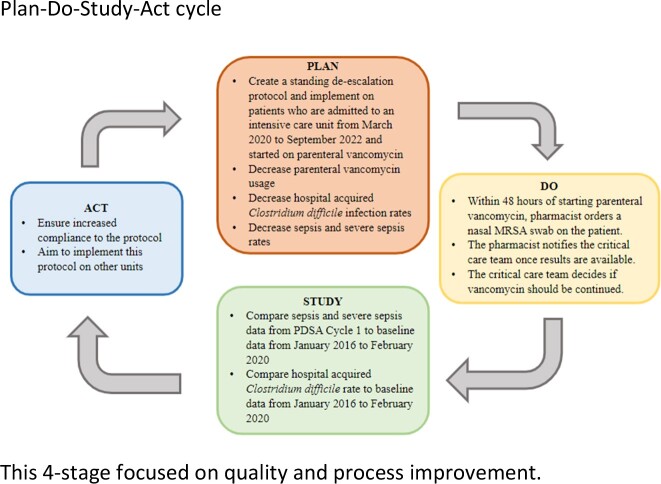

**Results:**

91/93 patients studied received MRSA nasal swabs. The average hospital stay was 12 days and average ICU stay was 5.6 days. The average vancomycin usage was 5.5 +/- 4.2 days, with discontinuation within 5.1 +/- 4 days of a negative MRSA swab, depending on the infection source. 2/93 (2.2%) restarted vancomycin. The average vancomycin usage (Monthly Days of Treatment/1000) prior to Protocol initiation was 79.63 and decreased to 67.17 with Protocol implementation. There was no significant impact on incidence of sepsis or *Clostridium difficile* infections.

Process metrics

**Figure:**
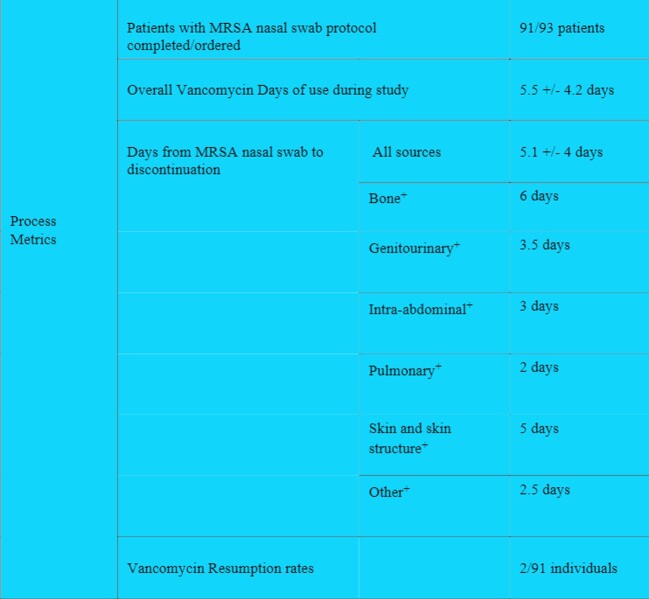

+Average days from MRSA nasal swab result to vancomycin discontinuation by suspected primary site of infection.

Outcome metrics

**Figure:**
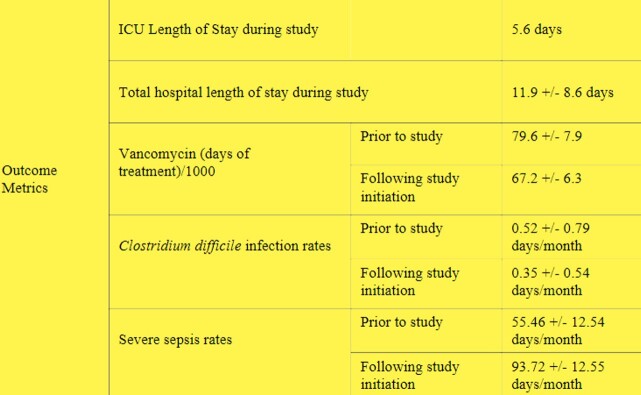

Study protocol was initiated in March 2020. Sepsis and severe sepsis rates were between the months of January 2016 to October 2022. C. difficile infection rate data was taken from January 2016 to December 2022.

**Conclusion:**

The primary goal of decreasing vancomycin usage with early MRSA nasal swab screening was successful. Standardized reevaluation of vancomycin usage after obtaining MRSA swab results led to discontinuation of vancomycin in 30/91 patients (32.9%) with 2/91 (2.2%) being restarted during the same admission. A linear regression model was applied to this data and supported our results. There was no significant impact on length of stay related to sepsis, or incidence of hospital acquired *Clostridium difficile* infection. Further study is warranted to see how the Protocol impacts these areas.

**Disclosures:**

**All Authors**: No reported disclosures

